# Magnetic and Electrical Characteristics of Nd^3+^ and Mn^2+^-Co-doped Calcium Molybdato-Tungstates Single Crystals

**DOI:** 10.1021/acsomega.3c05253

**Published:** 2023-10-02

**Authors:** Bogdan Sawicki, Elżbieta Tomaszewicz, Tadeusz Groń, Monika Oboz, Joachim Kusz, Marek Berkowski

**Affiliations:** † Institute of Physics, University of Silesia, Katowice 40-007, Poland; ‡ Faculty of Chemical Technology and Engineering, Department of Inorganic and Analytical Chemistry, West Pomeranian University of Technology, Szczecin 70-310, Poland; § Institute of Physics, Polish Academy of Sciences, Warszawa 02-668, Poland

## Abstract

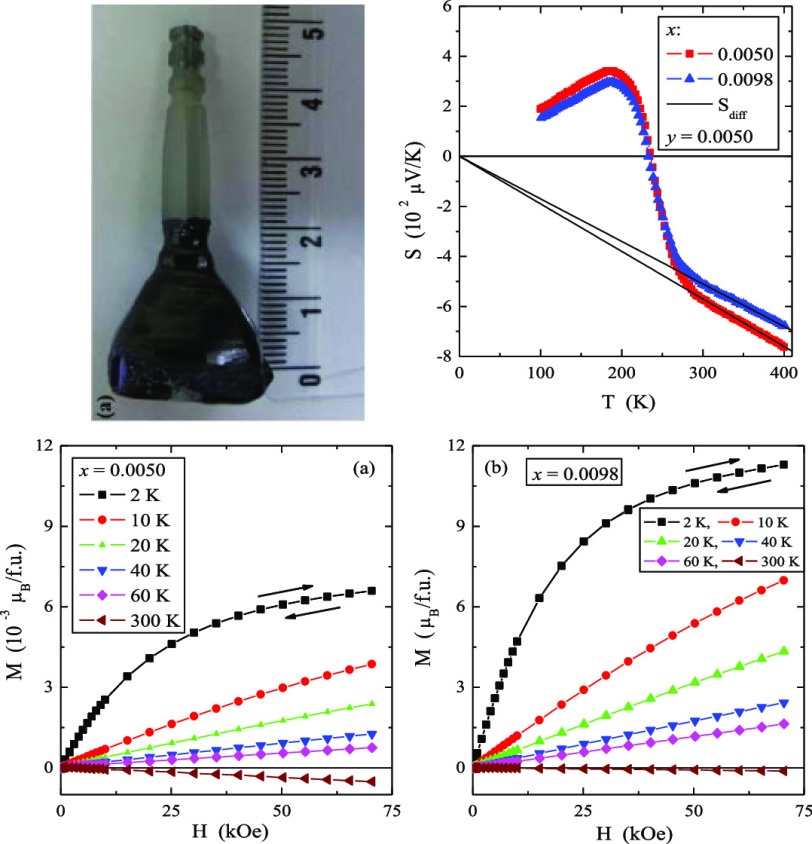

Single crystals of Ca_1–3*x*–*y*_Mn_*y*_□_*x*_Nd_2*x*_(MoO_4_)_1–3*x*_(WO_4_)_3*x*_ molybdato-tungstates (□ denotes vacant sites) with a scheelite-type structure have been successfully grown by the Czochralski technique in an inert atmosphere. This paper presents the results of structural, optical, magnetic, and electrical properties, as well as the broadband dielectric spectroscopy measurements of single crystals with different Nd^3+^ ion concentrations, *i.e.*, when *x* = 0.0050 or *x* = 0.0098, and with constant content of Mn^2+^ ions, *i.e.*, when *y* = 0.0050. Our magnetic studies have shown that substitution of diamagnetic Ca^2+^ ions in the CaMoO_4_ matrix with paramagnetic Nd^3+^ ones with a content not exceeding 0.02 and having a screened 4f-shell revealed a significant effect of orbital diamagnetism and Van Vleck’s paramagnetism. Both single crystals have revealed residual electrical conductivity without an intrinsic region and a change of sign of the Seebeck coefficient at ca. 230 K. Dielectric spectroscopy measurements have shown constant values of relative permittivity (ε_r_ ∼ 8) and loss tangent (tan δ ∼ 0.01) both up to 400 K and up to 1 MHz, as well as the Fermi energy (∼0.04 eV) and the Fermi temperature (∼500 K) determined for both crystals from the diffusion component of thermopower. These results suggest the presence of shallow acceptor and donor levels in the studied crystals.

## Introduction

Divalent metal molybdates and tungstates with the tetragonal scheelite-type structure (space group *I*4_1_/*a*), both undoped as well as activated with d- or f-electron metal ions, are important materials because of their interesting luminescent and magnetic properties. They are already used as, *e.g.*, efficient phosphors,^1,2^ solid state lasers^3^ scintillators,^4,5^ microwave dielectrics,^6,7^ and catalysts.^8^ The combination of several doping ions (called co-doping) is an increasingly popular and promising way of obtaining new materials with even more interesting properties and wide industrial applications, *e.g.*, ceramics such as CaSO_4_/Dy^3+^, Mn^2+^, or MgB_4_O_7_/Tb^3+^, Mn^2+^. These micropowders, when irradiated with γ-radiation, show 2–4 times more intensive thermoluminescence than the materials currently used in ionizing radiation dosimeters.^9,10^

Calcium molybdate (powellite, CaMoO_4_) and calcium tungstate (scheelite, CaWO_4_), as members of the scheelite family, are highly ionic compounds with a small contribution of covalent bonding. Band gaps determined from the band dispersion calculations indicated that both compounds are direct band gap insulators, with values of 3.41 and 4.09 eV for CaMoO_4_ and CaWO_4_, respectively.^11^

Our previous studies of calcium molybdate nanomaterials doped with Mn^2+^ ions^12^ and calcium molybdato-tungstate microcrystals doped with Mn^2+^^13^ or Co^2+^ and Gd^3+^^14^ ones have generally shown that they are nonconductive diamagnets or paramagnetics with a spin-only contribution for molybdates and a spin–orbit coupling for molybdato-tungstates. In these materials, a relatively low value of dielectric permittivity at low frequencies was observed, which is characteristic for space charge polarization. In turn, our studies of cadmium molybdate single crystals doped with Nd^3+^,^15^ Dy^3+^,^16^ or Eu^3+^ ions^17^ and microcrystals doped with Dy^3+^ ones,^18,19^ as well as molybdato-tungstate single crystals doped with Yb^3+^ ions,^20^ revealed semiconducting properties when single crystals contained Nd^3+^, Eu^3+^, and Yb^3+^ ions and nonconductive for micropowders containing Dy^3+^ ones. In all these materials based on cadmium molybdate or cadmium molybdato-tungstate, a paramagnetic state was observed in the temperature range of 2–300 K. Interesting dielectric properties have been discovered in CdMoO_4_/Eu^3+^ single crystals above room temperature, where the dipole relaxation process was accompanied by colossal dielectric permittivity.^17^ This unexpected effect was because the width of europium multiplets becomes comparable to thermal energy during heating. Also, our research on microcrystalline scheelite-type lead tungstates doped with Pr^3+^^21^ and Nd^3+^ ions,^22^ as well as lead molybdato-tungstates doped with Gd^3+^ ions,^23^ showed the nonconductive and paramagnetic properties of these doped materials. However, lead molybdato-tungstate single crystals doped with Nd^3+^ ions exhibited n-type semiconducting properties in the intrinsic region and a Fermi energy of 0.04 eV (derived from the diffusion component of thermopower), characteristic of shallow donor levels.^24^ Summarizing our results on a wide group of scheelite-type materials (micro- or nanopowders) codoped with divalent d-electron metal ions and trivalent rare-earth metal ones, it can be stated that paramagnetism is dominant everywhere, mainly due to the screening of electrons in the 4f subshells of RE^3+^ ions. For the samples poorer in magnetic ions, magnetic contributions independent of temperature, such as orbital diamagnetism or Van Vleck paramagnetism in nonmetals, played an important role. Generally, doped scheelite-type single crystals for such matrices as CdMoO_4_, CdMoO_4_/WO_4_, and PbMoO_4_/WO_4_ show semiconducting properties. Dipole relaxation processes and colossal dielectric permittivity were only observed for a CdMoO_4_/Eu^3+^ single crystal.

Recently, the solid solution of Ca_1–3*x*–*y*_Mn_*y*_□_*x*_Nd_2*x*_(MoO_4_)_1–3*x*_(WO_4_)_3*x*_ (*x* = 0.0050, 0.0098, 0.0192, 0.0283, 0.0370, 0.0455, 0.0839, 0.1430, 0.1667, 0.2000, and *y* = 0.0200) with very similar Mn^2+^ and Nd^3+^ concentrations as the single crystals under study was synthesized by the solid-state reaction method and studied.^25^ These studies revealed a scheelite-type structure of obtained micropowders, a direct band gap in the center of the Brillouin zone changing nonlinearly with an increase in the content of Nd^3+^ ions in the range of 3.70–3.83 eV, a significant effect of orbital diamagnetism and Van Vleck’s paramagnetism for samples poorer in neodymium ions, a paramagnetic behavior with long-range ferrimagnetic (FIM) and short-range antiferromagnetic (AFM) interactions, weak n-type electrical conductivity characteristic for insulators, an extrinsic region with low activation of 0.004 eV below 300 K and an intrinsic one with stronger activation of 1.0 eV above 300 K, and small values of both relative permittivity (∼12) and loss tangent (∼0.01), which were weakly dependent on temperature and frequency.^25^

In this study, Ca_1–3*x*–*y*_Mn_*y*_□_*x*_Nd_2*x*_(MoO_4_)_1–3*x*_(WO_4_)_3*x*_ single crystals (*x* = 0.0050 and 0.0098; *y* = 0.0050), obtained by the Czochralski method, were systematically investigated and compared with the microcrystalline materials of the same chemical formula. The study of poorly conductive and highly magnetically diluted single crystals and the impact of technology on their properties is a novelty of this work.

## Experimental Details

### Crystal Growth and Chemical Analysis of Single Crystals

**Figure 1:**
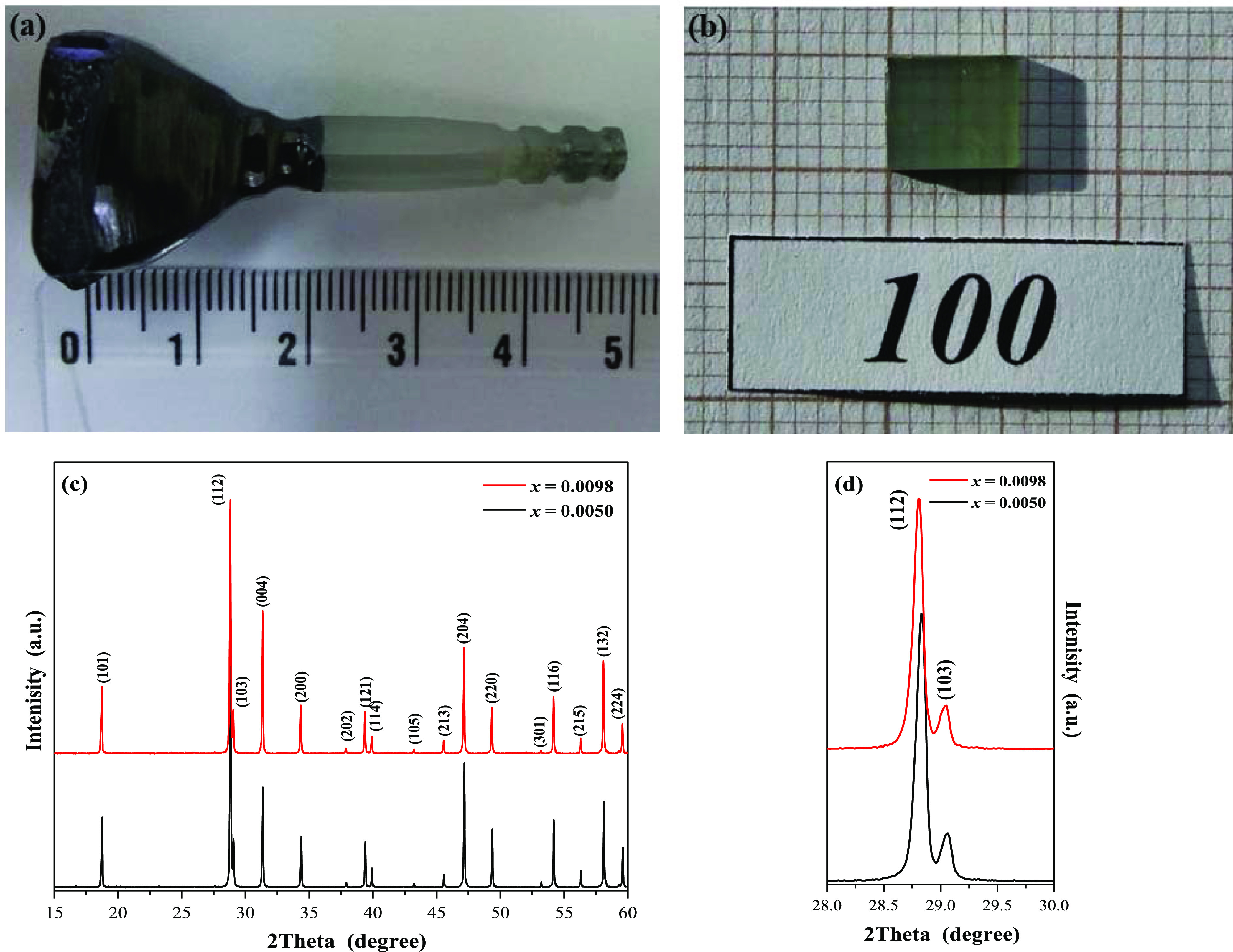
Image of the CMNMWO single crystal (*x* = 0.0050 and *y* = 0.0050) (a); image of a plate cut from this single crystal along the [100] direction (b); XRD patterns of both powdered CMNMWO single crystals in the range of 2Θ from 15 to 60° (c); (112) and (103) diffraction lines in the range of 2Θ from 28 to 30° (d).

Single crystals of Ca_1–3*x*–*y*_Mn_*y*_□_*x*_Nd_2*x*_(MoO_4_)_1–3*x*_(WO_4_)_3*x*_ solid solution (*x* = 0.0050 or 0.0098; *y* = 0.0050 and □ denotes vacant sites, labeled later as CMNMWO) were successfully grown by the Czochralski method in an inductively heated iridium crucible in nitrogen as a protective atmosphere. The starting materials for the crystallization processes of both single crystals were: CaCO_3_ (99.95%, VWR Chemicals Prolabo), MnO (99.99%, Alfa Aesar), Nd_2_O_3_ (99.99%, Alfa Aesar), MoO_3_ (99.95%, Alfa Aesar), and WO_3_ (99.95%, Alfa Aesar). The stoichiometric amounts of initial reactants were ground homogeneously in an agate mortar, pressed into cylindrical pellets under pressure of 200 kPa, and heated at 800 °C for 12 h in the air before melting in an iridium crucible. Then, the microcrystalline materials were heated to a temperature of ∼50 °C higher than their melting point. Thus, the obtained melts were maintained under these conditions for 2 h. Next, the temperature was decreased to the appropriate crystallization point. The single crystals of calcium molybdato-tungstates codoped with Nd^3+^ and Mn^2+^ ions were grown on the [001]-oriented seeds prepared from a single crystal of CaMoO_4_. The as-grown crystals were dark blue in color (Figure 1a). They were not subjected to additional oxidation processes in an oxygen or air atmosphere. After accurate orientation by the X-ray diffraction (XRD) method, the CMNMWO single crystals were cut along the [100] and [001] crystallographic planes, and then plates of dimensions of ∼5 × 6 × 1 mm were cut off (Figure 1b). Such-prepared samples were used for the optical, magnetic, and dielectric studies. For structural studies, small pieces of diameter less than 0.1 mm were cut off and selected under a polarizing microscope. The contents of calcium, manganese, neodymium, molybdenum, and tungsten in the CMNMWO single crystals were determined by an inductively coupled plasma mass spectrometry (ICP–MS) technique. Samples of both crystals were previously dissolved in a dilute hydrochloric acid solution. The contents of metallic elements were found as CMNMWO (*x* = 0.0050, *y* = 0.0050) single crystal—Ca 19.35(4) mas % (cal. 19.42 mas %); Mn 0.13(1) mas % (cal. 0.14 mas %); Nd 0.67(2) mas % (cal. 0.71 mas %); Mo 46.64(6) mas % (cal. 46.72 mas %); W 1.39(2) mas % (cal. 1.36 mas %); the CMNMWO (*x* = 0.0098, *y* = 0.0050) single crystal—Ca 18.87(4) mas % (cal. 18.94 mas %); Mn 0.12(1) mas % (cal. 0.13 mas %); Nd 1.33(4) mas % (cal. 1.38 mas %); Mo 45.49(7) mas % (cal. 45.57 mas %); and W 2.60(3) mas % (cal. 2.65 mas %). The determined contents of Ca, Mn, Nd, Mo, and W in both CMNMWO single crystals closely corresponded to the proposed chemical formulas, *i.e.*, when *x* = 0.0050 or *x* = 0.0098 and *y* = 0.0050. The density of both CMNMWO crystals was determined using a Quantachrome Instruments Ultrapycnometer (model Ultrapyc 1200 e, USA). Nitrogen (purity 99.99%) was applied as a pycnometric gas. The values of density were found to be 4.34(2) g cm^–3^ (*x* = 0.0050) and 4.35(2) g cm^–3^ (*x* = 0.0098). Small pieces of both CMNMWO single crystals obtained after cutting out the plates were ground in an agate mortar. The obtained microcrystalline powders were examined by a powder XRD method using an EMPYREAN II diffractometer (PANalytical) and Cu K_α1,2_ radiation (λ = 1.5418 Å). XRD patterns were collected in the 10–100° 2Θ range with a scanning step of 0.013° and analyzed by HighScore Plus 4.0 software. Lattice constants were calculated using DICVOL04 software.^26^Figure 1c,d shows the XRD patterns of powdered CMNMWO single crystals in different 2Θ ranges. All observed diffraction lines were successfully indexed to a pure tetragonal, body-centered scheelite-type structure with space group *I*4_1_/*a* (JCPDs No. 04-013-6763).

### Methods

A SuperNova kappa diffractometer, equipped with a Mo Kα X-ray tube and Atlas CCD detector (Agilent Technologies), was used for the XRD measurements, which were performed at 293(1) K. The CrysAlis^Pro^^27^ program was used for the collection of the data as well as for determination and refinement of the unit cell parameters from about 4000 reflections. Integrations of the collected data were also performed using CrysAlis^Pro^.^27^ SHELXL-97 program^28^ was used to refine the structure of both single crystals. The positions of O atoms and the anisotropic displacement parameters of all atoms were refined.

Ultraviolet and visible (UV–vis) diffuse reflectance spectroscopy was realized with a JASCO-V670 (Jasco International Co., Tokyo, Japan) spectrophotometer equipped with an integrating sphere. The spectra were recorded in the range of 200 to 1000 nm with a scanning rate of 0.5 nm.

The static (DC) magnetic susceptibility was measured in the temperature range of 2–300 K and recorded in both zero-field-cooled (ZFC) and field-cooled (FC) modes. Magnetization isotherms were measured at 2, 10, 20, 40, 60, and 300 K by using a Quantum Design MPMS-XL-7AC SQUID magnetometer (Quantum Design, San Diego, CA, USA) in applied external fields up to 70 kOe. The effective magnetic μ_eff_ moment was determined using the eq 1.^29,30^
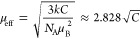
1where *k* is the Boltzmann constant, *N*_A_ is the Avogadro number, μ_B_ is the Bohr magneton, and *C* is the molar Curie constant. The effective number of Bohr magnetons *p*_eff_ was calculated from the eq 2
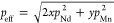
2where (31) for Nd^3+^ (*J* = 9/2, *L* = 6, *S* = 3/2, *g* = 8/11, basic term ^4^I_9/2_) and Mn^2+^ (*J* = 5/2, *L* = 0, *S* = 5/2, *g* = 2, basic term ^6^S_5/2_) ions with 4f^3^ and 3d^5^ electronic configuration, respectively.

Electrical conductivity, σ(*T*), was measured by the DC method using a KEITHLEY 6517B electrometer/high-resistance meter (Keithley Instruments, LLC, Solon, OH, USA). The thermoelectric power *S*(*T*), *i.e.*, the Seebeck coefficient, was measured in the temperature range of 100–400 K with the help of a Seebeck Effect Measurement System (MMR Technologies, Inc., San Jose, CA, USA).

Broadband dielectric spectroscopy measurements were performed using a LCR HITESTER HIOKI 3532-50 (Hioki E.E. Corporation, Nagano, Japan) within the temperature range of 76–400 K and in the frequency range from 5 × 10^2^ to 1 × 10^6^ Hz. The electrode area and thickness were 5 mm^2^ and 1.0 mm, respectively. Relative dielectric permittivity was determined from the eq 3
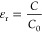
3where *C*_0_ is the capacity of the empty capacitor and
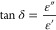
4where ε″ and ε′ are the imaginary and real parts of complex dielectric permittivity, respectively. The electrical and thermal contacts were made with a silver lacquer mixture (Degussa Leitsilber 200, Degusta Gold and Silber, Munich, Germany).

## Results and Discussion

### Crystal Structure

The XRD studies revealed that both single crystals belong to tetragonal symmetry and crystallize with scheelite-type structure in the *I*4_1_/*a* space group, similarly to some divalent molybdates and tungstates, *i.e.*, AMo(W)O_4_ (A = Ca, Sr, Ba, and Pb).^10^ The unit cell parameters of the CMNMWO crystal (*x* = 0.0050; *y* = 0.0050) are as follows: *a* = *b* = 5.22766(10) Å and *c* = 11.4330(3) Å. The *R* value is equal to 0.0149. In the case of the CMNMWO single crystal (*x* = 0.0098; *y* = 0.0050), the lattice constants are as follows: *a* = *b* = 5.23207(12) and *c* = 11.4424(5) Å. The *R* value is equal to 0.0164. The most important crystallographic data for both single crystals are collected in Tables S1–S8. The calculated lattice parameters of both crystals increased with the increase in Nd^3+^ ions concentration. This is not a typical behavior because bigger Ca^2+^ ions in the CaMoO_4_ matrix were simultaneously substituted by much smaller Nd^3+^ and Mn^2+^ ones (*r* (Ca^2+^) = 1.12 Å > *r* (Nd^3+^) = 1.053 Å > *r* (Mn^2+^) = 0.96 Å for CN = 8).^32^ In scheelite-type molybdates and tungstates when *r* (A^2+^) > 0.9 Å, molybdenum and tungsten ions are tetrahedrally coordinated by O^2–^ ones, and their ionic radii are very similar, *i.e.*, they are 0.41 and 0.42 Å, respectively.^32^ For this reason, no significant changes in the lattice parameters of both CMNMWO single crystals should be observed when Mo^6+^ ions are substituted by W^6+^ ones. The crystal lattice expansion observed for CMNMWO single crystals is also visible after grinding these samples. With the increase of Nd^3+^ content, all diffraction lines observed in the XRD patterns of powder materials shifted toward the lower angle direction (Figure 1c). In order to show the changes of position of peaks clearly, the enlarged portions of the XRD patterns from 28 to 30° 2Θ with (112) and (103) diffraction lines are presented in Figure 1d. The calculated lattice parameters of powdered crystals are as follows: CMNMWO (*x* = 0.0050) *a* = *b* = 5.22731(10) and *c* = 11.4296(4) Å; CMNMWO (*x* = 0.0098) *a* = *b* = 5.23096(11) and *c* = 11.4396(6) Å.

### Optical Properties

The optical properties of CMNMWO single crystals along both crystallographic directions were investigated at room temperature by using the UV–vis diffuse reflectance spectroscopy method. This technique enables the determination of such important optical parameters of materials as the band gap energy, absorption coefficient, and others. The theory which makes possible the use of diffuse reflectance spectra for solids was proposed by Kubelka and Munk.^33^

**Figure 2:**
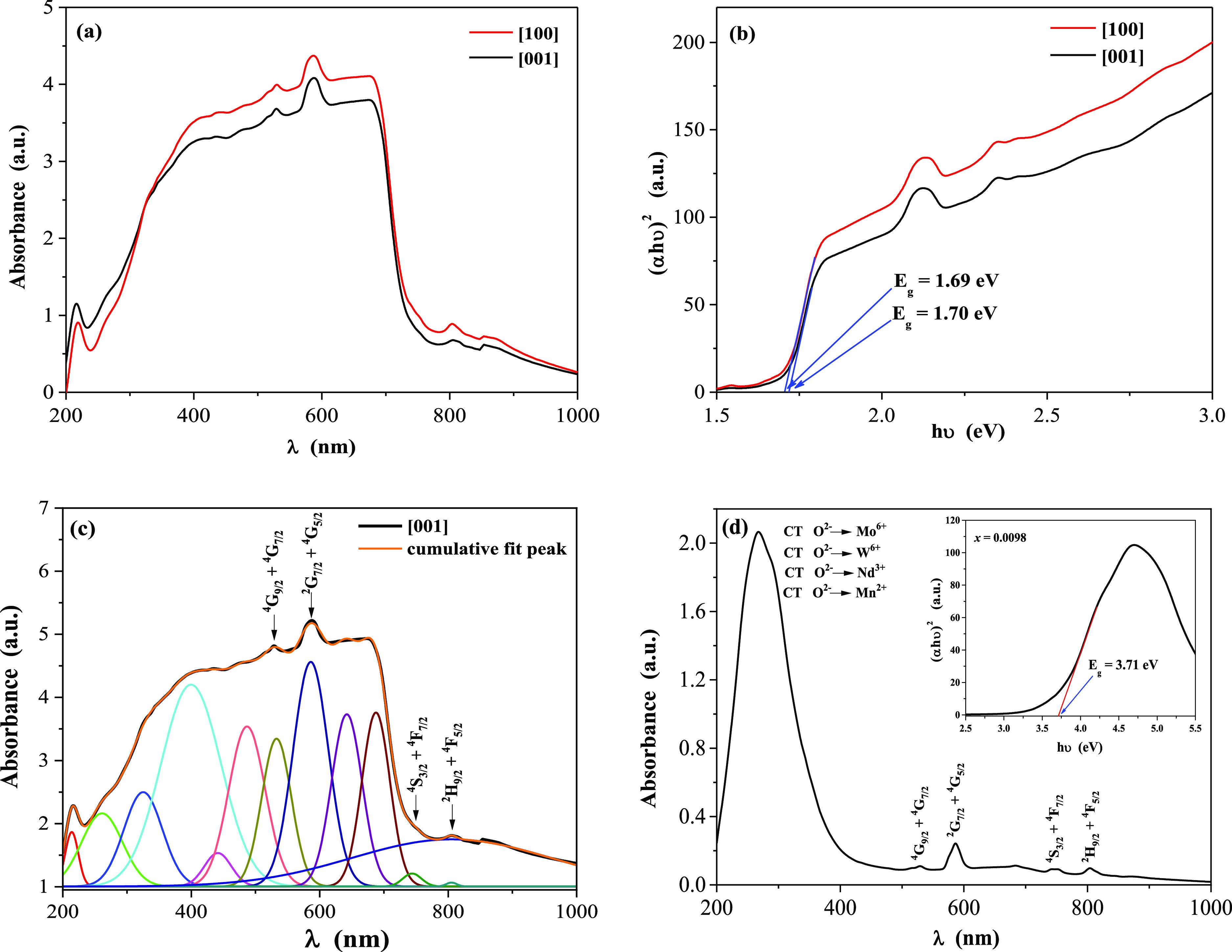
(a) UV–vis absorption spectra of the CMNMWO single crystal (*x* = 0.0098) for [100] and [001] crystallographic directions; (b) plot of (α*h*ν)^2^ vs *h*ν of CMNMWO single crystal (*x* = 0.0098) for both crystallographic directions with determined band gap energy (*E*_g_) values; (c) Gaussian fit of the UV–vis absorption spectrum of the CMNMWO single crystal (*x* = 0.0098) for the [001] crystallographic direction; (d) UV–vis absorption spectrum of the powdered CMNMWO single crystal (*x* = 0.0098); insert—plot of (α*h*ν)^2^ vs *h*ν for this sample and determined band gap energy.

According to this method, the reflectance spectra can be converted into absorption ones using the eq 5.^33^
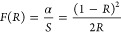
5where *F*(*R*) is the Kubelka–Munk approach, *R* is the reflectance, α is the absorption coefficient, and *S* is the scattering factor, which is wavelength-independent. The optical band energy gap (*E*_g_) is related to the absorbance and photon energy in the eq 6 proposed by Tauc^34,35^
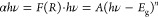
6where *h*ν is the photon energy, *A* is an energy-independent constant characteristic of a material, and *n* is a constant that can take different values depending on the nature of the electronic transition. The permitted direct, forbidden direct, permitted indirect, and forbidden indirect transitions take place when *n* = 1/2, 3/2, 2, and 3, respectively.^34,35^ According to the literature, scheelite-type materials such as CaMoO_4_ and CaWO_4_ show an optical spectrum regulated by the direct absorption process, *i.e.*, when *n* = 1/2.^10^ In the low-energy region, the absorption edge (α*h*ν)^2^ changed linearly with the energy of the photon. Thus, in the high-energy region, the absorption spectrum deviated from the straight-line plot. This straight-line behavior in the low-energy region was considered the main evidence of a direct optical bandgap. Figure 2 a shows the UV–vis absorbance spectra of a CMNMWO single crystal when *x* = 0.0098 in the wavelength from 200 to 1000 nm. The intense and very broad band is observed in the spectral range of 300–700 nm for this sample. A similar broad band is also observed for the CMNMWO single crystal with a lower concentration of Nd^3+^ ions (not shown here). In this spectral range, the following charge transfer bands occur: O^2–^ → Mo^6+^, O^2–^ → Mo^5+^ present in small amounts in as-grown CMNMWO single crystals, O^2–^ → W^6+^, O^2–^ → Nd^3+^ as well as O^2–^ → Mn^2+^.^36–38^ In the visible range, the bands associated with 4f–4f transitions of Nd^3+^ are also observed.^39,40^Figure 2 c shows the UV–vis spectrum of a CMNMWO single crystal (*x* = 0.0098) for the [001] crystallographic direction deconvoluted into Gaussian components with the assignment of bands for Nd^3+^ ions in the visible region.

The values of the direct band gap of CMNMWO single crystals were obtained by extrapolating a linear region of the (α*h*ν)^2^ curve obtained for both crystals as well as for [100] and [001] crystallographic directions to the photon energy axis (Figure 2b). The direct band gaps of both CMNMWO single crystals were found to be when *x* = 0.0050 and *y* = 0.0050 and for [100] crystallographic direction −1.73 eV and for [001] direction −1.72 eV; when *x* = 0.0098 and *y* = 0.0050 and for [100] −1.69 eV and for [001] −1.70 eV (Figure 2b). The observed values of *E*_g_ are slightly lower for the single crystal, where the concentration of Nd^3+^ ions is higher. Slightly different values of the energy gap determined for both crystallographic directions of each single crystal show that they do not demonstrate anisotropy of the optical properties. For this reason, magnetic and electrical studies of both single crystals were performed along one [001] crystallographic direction only. Figure 2d shows the UV–vis absorption spectrum of the ground in an agate mortar of the CMNMWO single crystal when *x* = 0.0098. This spectrum is different compared to the spectra of single crystal samples. The charge transfer band of O^2–^ → Mo^5+^ is not observed for powdered samples. Their *E*_g_ values were found to be 3.73 eV (*x* = 0.0050 and *y* = 0.0050) as well as 3.71 eV (*x* = 0.0098 and *y* = 0.0050, Figure 2c insert), and they are more than twice as high as for single crystals.

### Magnetic Properties

**Figure 3:**
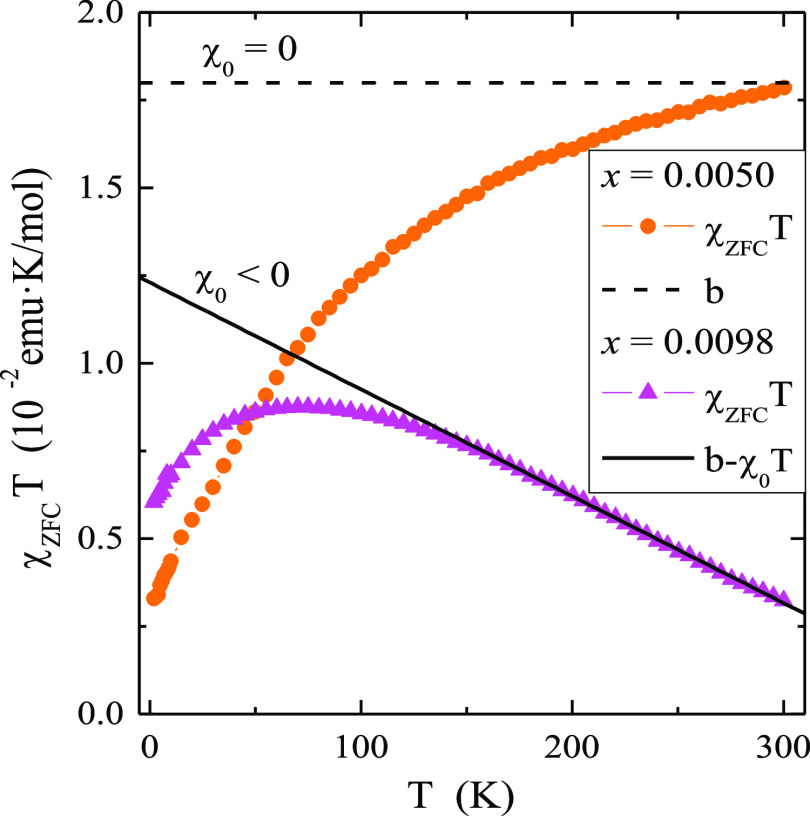
Product χ_ZFC_*T* vs temperature *T* of CMNMWO single crystals for *x* = 0.0050 and *x* = 0.0098 with constant content *y* = 0.0050 ([001] crystallographic direction). The solid and dashed lines, χ*T*(*T*), indicate Curie–Weiss behavior. χ_0_ is the temperature-independent contribution of magnetic susceptibility.

**Figure 4:**
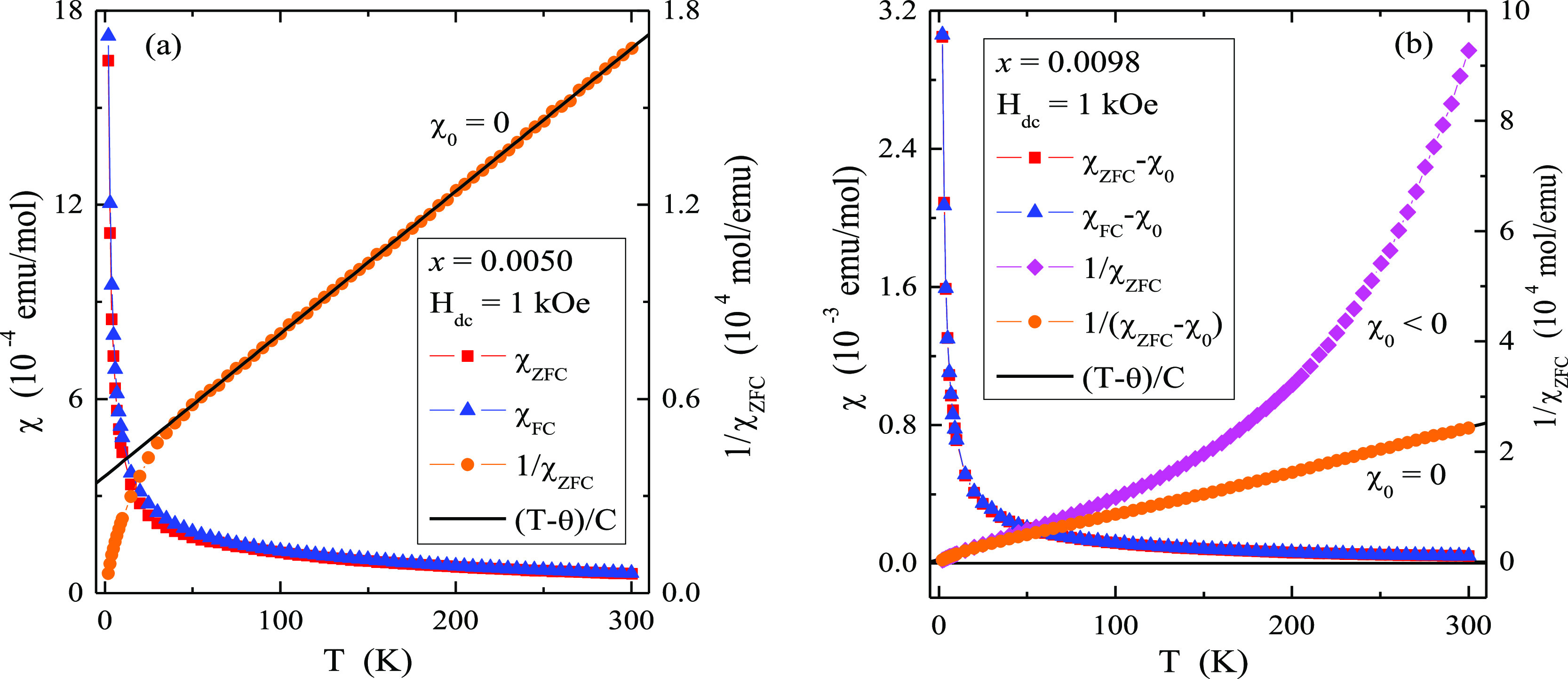
Magnetic susceptibility χ_ZFC_, χ_FC_, 1/χ_ZFC_, χ_ZFC_ – χ_0_, χ_ZFC_ – χ_0_, 1/(χ_ZFC_ – χ_0_) and (*T* – θ)/*C* vs temperature *T* of CMNMWO single crystals: (a) *x* = 0.0050 and *y* = 0.0050, (b) *x* = 0.0098 and *y* = 0.0050 recorded at *H*_dc_ = 1 kOe ([001] crystallographic direction).

**Figure 5:**
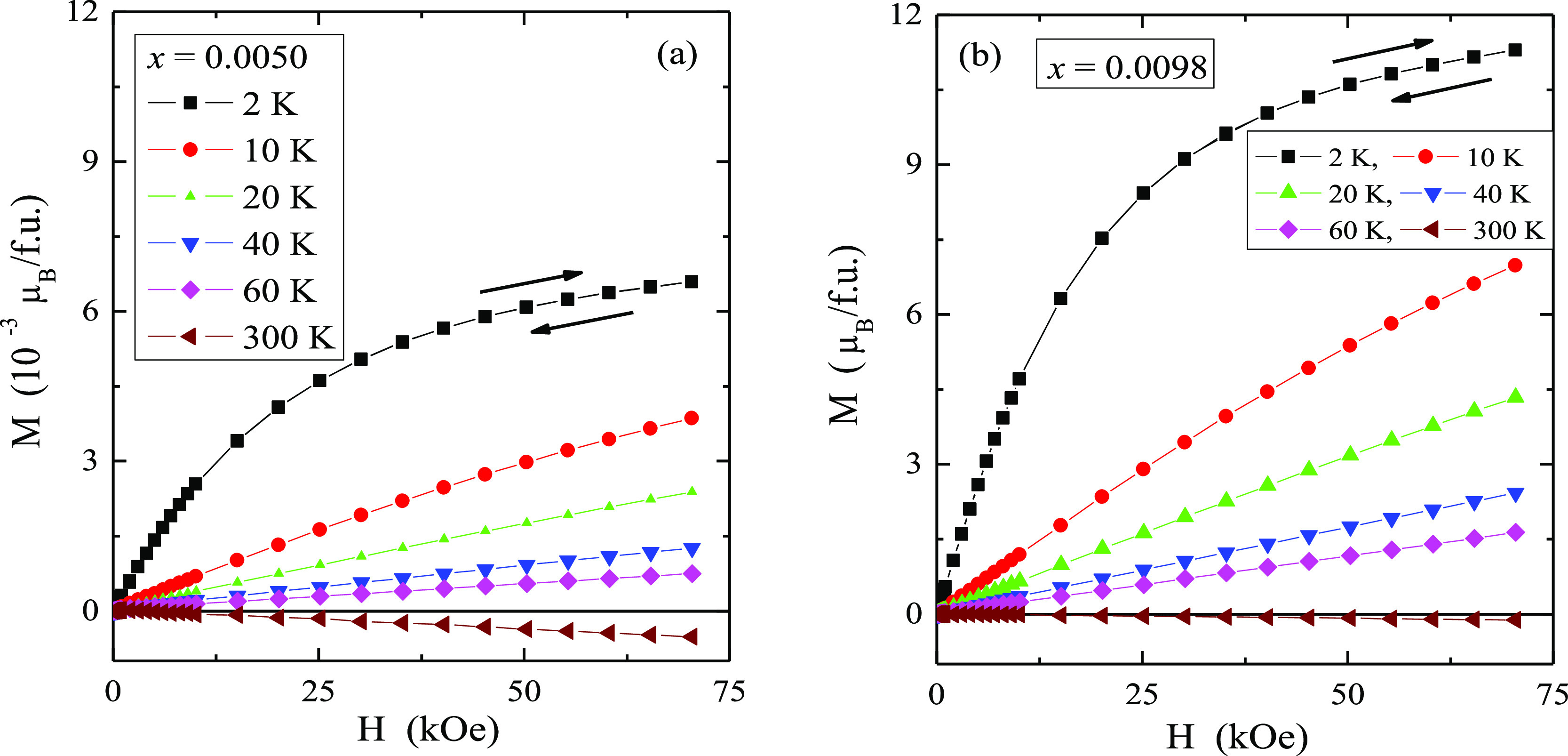
Magnetization *M* vs magnetic field *H* of CMNMWO single crystals: (a) *x* = 0.0050 and *y* = 0.0050, (b) *x* = 0.0098 and *y* = 0.0050, recorded at 2, 10, 20, 40, 60, and 300 K. A run of the magnetic field is indicated by arrows ([001] crystallographic direction).

The results of magnetic measurements of the CMNMWO single crystals are depicted in Table 1 and Figures 3–5. Due to the low content of magnetic neodymium and manganese ions, which did not exceed 0.02 and 0.005, respectively, it was necessary to estimate the temperature-independent magnetic contributions (χ_0_), as they affect the magnetic parameters of the single crystals under study. In general, these contributions significantly change the value of the magnetic susceptibility in the Curie–Weiss region by the factor χ_0_, which represents all temperature-independent susceptibilities.^41^ The χ_0_ factor was determined from the linear function χ*T*(*T*) in the Curie–Weiss region from the eq 7.^24^
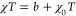
7where *b* is the intercept, and χ_0_ is the slope. The dependencies of product χ_ZFC_·*T*(*T*) from the measurement and the asymptotes determined from eq 7 in the Curie–Weiss region are shown in Figure 3, and parameters *b* and χ_0_ are shown in Table 1.

**Table 1:** Magnetic Parameters of CMNMWO Single Crystals^a^

*x*	*y*	*C* (emu K/mol)	θ (K)	μ_eff_ (μ_B_/f.u.)	*p*_eff_	*M*_0_ (μ_B_/f.u.)	χ_0_ (emu/mol)	*b* (emu K/mol)
0.0050	0.0050	0.0227	–82	0.426	0.553	0.0066	0	0.018
0.0098	0.0050	0.0125	–5	0.317	0.659	0.0113	–3.045 × 10^–^^5^	0.0123

a *x* is half of the content of neodymium ions in the crystal, *y* is the content of manganese ions in the crystal, *C* is the Curie constant, θ is the Curie–Weiss temperature, μ_eff_is the effective magnetic moment, *p*_eff_ is the effective number of Bohr magnetons, *M*_0_ is the magnetization at 2 K and 70 kOe, and χ_0_ and *b* are the slope and the intercept of the linear χ_ZFC_*T*(*T*) function, respectively.

Figure 3 shows the compensation of Van Vleck’s paramagnetism and orbital diamagnetism (χ_0_ = 0) for a single crystal with *x* = 0.0050, while orbital diamagnetism (χ_0_ < 0) dominates for a single crystal with *x* = 0.0098. This means that for a larger amount of neodymium ions in the sample, Van Vleck paramagnetism weakens. Both single crystals shown in Figure 4 are paramagnetics within the temperature range of 2–300 K, indicating the Curie–Weiss behavior and FIM long-range interactions evidenced by a deviation of the 1/χ_ZFC_(*T*)-curve downward from its linear part as well as the short-range AFM interactions visible in negative values of the Curie–Weiss temperature (Table 1).

No splitting between ZFC and FC magnetic susceptibilities suggests no spin frustration. The effective magnetic moment, μ_eff_, estimated from eq 1 is much smaller compared to the effective number of Bohr magnetons, *p*_eff_, especially for a sample with *x* = 0.0098. While for the sample with the content of *x* = 0.0050 the difference is not so large, which can be explained by the appearance of manganese ions in the oxidation state higher than +2, for the sample with the content of *x* = 0.0098 the difference is already 50%. Such a large difference cannot be explained only by the change in the oxidation state of both magnetic ions. In this case, the reason may be the strong influence of orbital diamagnetism, spin–orbit coupling, and the competition of FIM and AFM interactions. In a solid solution with the same chemical formula as the studied single crystals, synthesized by a high-temperature solid-state reaction with a content of 0.0050 ≤ *x* ≤ 0.2000 and *y* = 0.0200,^25^ it was observed that for samples poorer in manganese, the μ_eff_ is slightly larger than the *p*_eff_, and for samples richer in manganese, vice versa. On the other hand, in the single crystals of the Pb_1–3*x*_□_*x*_Nd_2*x*_(MoO_4_)_1–3*x*_(WO_4_)_3*x*_ solid solution (*x* = 0.0010 and 0.0050),^24^ it was observed that the values of the effective magnetic moment (μ_eff_) are much higher than the effective number of Bohr magnetons (*p*_eff_).

This difference was explained by the fact that some molybdenum ions may be in an oxidation state lower than +6, contributing to an increase in the net magnetic moment.

The results of the magnetic moment measurements of CMNMWO single crystals are shown in Table 1 and Figure 5. Magnetic isotherms do not have hysteresis, coercive field, and remanence. With the increase of the magnetic field and the content of neodymium ions in the sample, magnetization increases, but does not reach saturation. Similar behavior was observed for polycrystalline samples^25^ of the same solid solution as the single crystals under study, as well as for single crystals of Pb_1–3*x*_□_*x*_Nd_2*x*_(MoO_4_)_1–3*x*_(WO_4_)_3*x*_ solid solution.^24^ The reason for this could be a magneto-crystalline anisotropy.

### Electrical Studies

**Figure 6:**
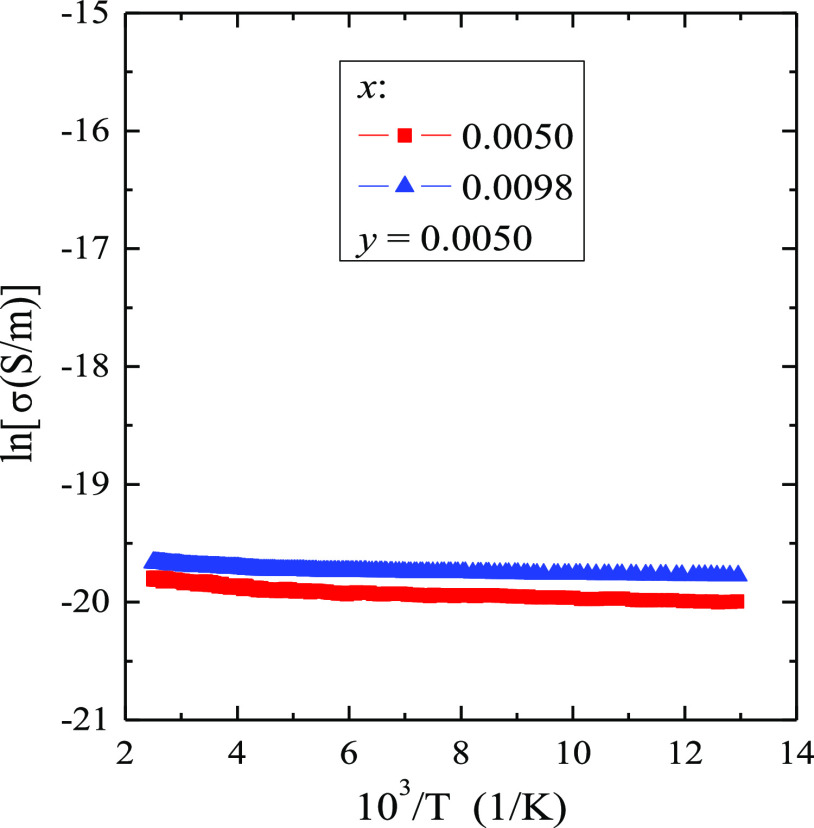
Electrical conductivity (ln σ) vs temperature *T* of CMNMWO single crystals ([001] crystallographic direction).

The results of the electrical conductivity measurements, σ(10^3^/*T*), of the CMNMWO single crystals showed residual electrical conductivity without intrinsic region in the temperature range of 77–400 K and the value of electrical conductivity at 400 K of only 2.7 × 10^–9^ S/m (Figure 6). This means that the increase in temperature does not generate new electric current carriers at impurity levels, even though the direct band gap values shown in Figure 2b are close to 1.70 eV.

**Figure 7:**
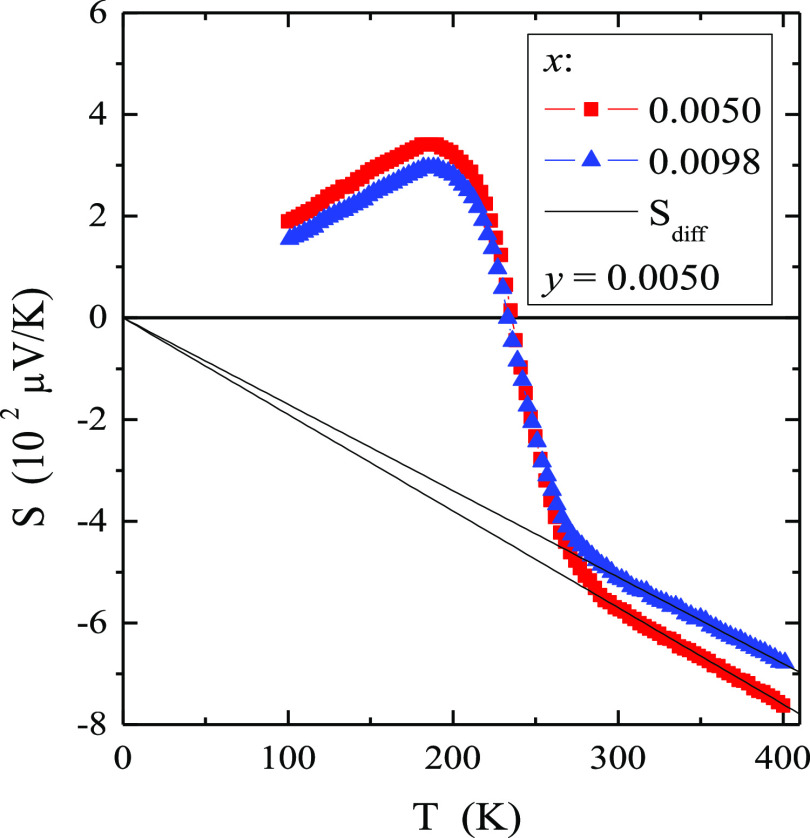
Thermoelectric power *S* vs temperature *T* of CMNMWO single crystals ([001] crystallographic direction).

No intrinsic region in the relation, σ(10^3^/*T*), was also observed among others in Ca_1–*x*_Mn_*x*_(MoO_4_)_0.50_(WO_4_)_0.50_ ceramics,^13^ Ca_1–*x*_Mn_*x*_MoO_4_ nanomaterials,^12^ CdRE_2_W_2_O_10_ tungstate mi crocrystals (RE = Y, Nd, Sm, and Gd–Er),^42^ and Ca_1–3*x*–*y*_Co_*y*_□_*x*_Gd_2*x*_(MoO_4_)_1–3*x*_(WO_4_)_3*x*_ molybdato-tungstate micropowders.^14^ Residual electrical conductivity of the p-type up to 230 K and the n-type above this temperature (Figure 7) in the extrinsic region seem to be related to the presence of a small amount of cationic and anion vacancies in the studied single crystals. Another explanation may be related to the fact that at a state of thermal equilibrium structural defects (*n*) are always present in the lattice even in a crystal which is ideal in other respects. A necessary condition for free energy minimalization gives *n* ≃ *N* exp (−*E*_V_/*kT*) for *n* ≪ *N*, where *N* is the number of atoms in the crystal, and *E*_V_ is the energy required to transfer the atom from the bulk of the crystal on its surface.^43^

The temperature dependence of the thermoelectric power *S*(*T*), shown in Figure 7, changes sign from positive to negative at 230 K. With residual electrical conductivity, the mechanism responsible for this process seems to be the transport of current carriers from the vacancy levels. It is known from the literature that the thermoelectric power in conventional metals consists of two different parts, *i.e.*, the diffusion contribution (*S*_diff_), which is proportional to temperature according to the Mott formula,^44^ and the phonon drag contribution (*S*_ph_), which is more complex and results from the transfer of phonon momentum to the electron gas. The *S*_ph_ component has an extreme in the range (θ_D_/10, θ_D_/2), where θ_D_ is the Debey temperature.^45^ The θ_D_ value estimated from the Debye formula^24^ is 349 K for both single crystals. The *S*(*T*) maximum observed below 230 K in Figure 7 indicates a transfer of phonon momentum to the electron gas. Our experimental dependence of the diffusion thermopower at high temperatures is a well-defined linear slope which extrapolates to (0, 0) (in Figure 7 marked by solid lines) according to the Boltzmann transport equation, as follows^44^
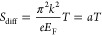
8Where *e* is the elementary charge, *E*_F_ is the Fermi energy, and *a* is an empirical slope. From eq 8, the Fermi energy, *E*_F_, can be written as follows
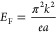
9

The values of *E*_F_ and Fermi temperature *T*_F_ (defined as *E*_F_/*k*) are summarized in Table 2. Compared to metals, *e.g.*, for pure copper: *E*_F_ = 7 eV and *T*_F_ = 8.19 × 10^4^ K^43^ and to non-metallic conductors, *e.g.*, for Cu_1–*x*_Ga_*x*_Cr_2_Se_4_ single crystals: *E*_F_ ∼ 0.3 eV and *T*_F_ ∼ 3 × 10^3^ K,^46^ as well as to Pb_1–3*x*_□_*x*_Nd_2*x*_(MoO_4_)_1–3*x*_(WO_4_)_3*x*_ single crystals: 0.036 ≤ *E*_F_ ≤ 0.048 eV and 418 ≤ *T*_F_ ≤ 557 K,^24^ it can be seen that the latter have very small values, such as the single crystals under study. The Fermi level in the studied single crystals with *E*_F_ = 0.04 eV compared to *E*_g_ = 1.7 eV means that the *E*_F_ is close to the border of the valence band, similar to the case of insulators. This means that the studied single crystals are in fact intrinsic semiconductors, in which the conductivity is half and half electron–hole. Therefore, an increase in electrical conductivity up to 400 K is not observed because electrons cannot increase their energy without jumping into the conduction band.

**Table 2:** Electrical Parameters of CMNMWO Single Crystals^a^

*x*	*y*	*A* (μV/K^2^)	*E*_F_ (eV)	*T*_F_ (K)	*E*_g_ (eV)
0.0050	0.005	–1.9	0.039	452	1.72
0.0098	0.005	–1.7	0.043	499	1.70

a *x* is half of the content of neodymium ions in the crystal, *y* is the content of manganese ions in the crystal, *a* is the slope of the linear *S*_diff_(*T*) diffusion function of thermopower, *E*_F_ is the Fermi energy, *T*_F_ is the Fermi temperature, and *E*_g_ is the band energy gap.

### Dielectric Properties

**Figure 8:**
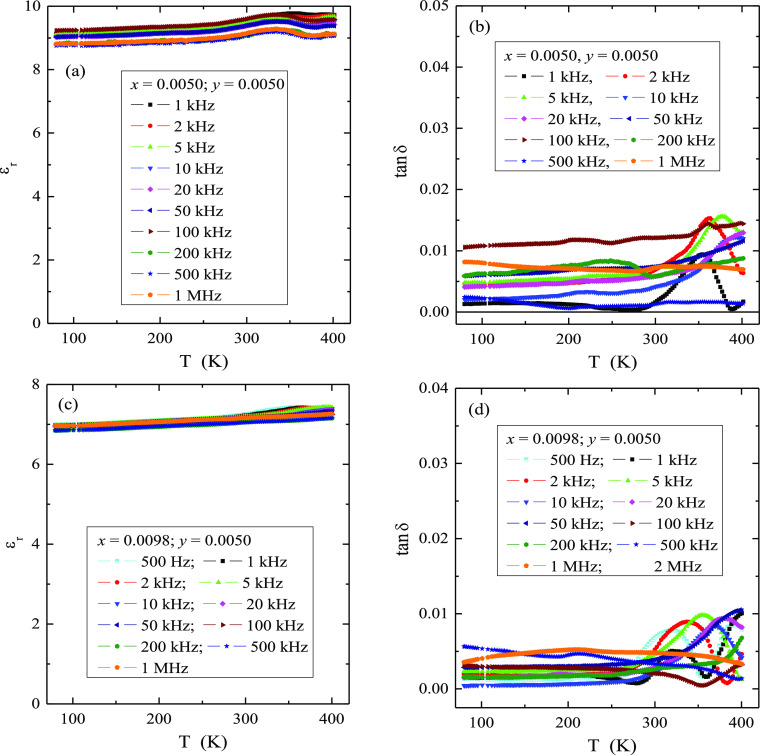
Relative dielectric permittivity, ε_r_ (a,c), and loss tangent, tan δ (b,d), vs temperature *T* of CMNMWO single crystals within the frequency range of 500 Hz–1 MHz ([001] crystallographic direction).

Figure 8 presents the temperature dependence of relative dielectric permittivity, ε_r_, and the loss tangent, tan δ, for the various electric field frequencies of the CMNMWO single crystals. As one can see, for each single crystal, the value of the permittivity remains almost independent of the frequency, and it only slightly increases with temperature. However, this increase is very minor, and, *e.g.*, for the single crystal when *x* = 0.0050, ε_r_ changes from 9.1 at 80 K up to 9.7 at 401 K (Figure 8a). Also, the values of tan δ of this single crystal remain small, usually ca. 0.01 for all temperatures and frequencies (Figure 8b). For the single crystal with *x* = 0.0098, ε_r_ (Figure 8c) and tan δ (Figure 8d) have slightly lower values compared to a single crystal with *x* = 0.0050. Microcrystalline materials with the same chemical formula^25^ have almost identical dependences of ε_r_ and tan δ as a function of temperature and frequency as the single crystals under study. This suggests that the technology of obtaining samples has no effect on the accumulation of electric charge or on energy loss. Impedance spectroscopy used to analyze the above results did not reveal the Cole–Cole semicircles (not presented here). This suggests that in the single crystals under study, no dipole relaxation processes were observed in the temperature range up to 400 K. Therefore, the accumulation of charge visible in the spectra may be caused by space charge polarization, as the studied single crystals are practically intrinsic semiconductors (insulators). Temperature dependences of ε_r_(*T*) and tan δ(*T*) do not also indicate an occurrence of dipole relaxation processes or energy losses at low frequencies induced by electric conduction, *i.e.*, so-called Joule-Lenz losses. Materials with such low energy loss can be successfully used in the fabrication of lossless capacitors. Similarly, a low energy loss was observed in vacancied lead tungstates with high Pr^3+^ ion content.^21^

## Conclusions

In this study, the effect of Ca^2+^ site substitution by Nd^3+^ and Mn^2+^ ions on structural, optical, magnetic, and dielectric properties in CMNMWO single crystals (where *x* = 0.0050 as well as *x* = 0.0098 and *y* = 0.0050) was studied. XRD analysis identified that both the CMNMWO crystals crystallize in a tetragonal scheelite-type structure (space group *I*4_1_/*a*). The direct optical band gap was found to be ∼1.70 eV for both single crystals and both crystallographic directions. The CMNMWO single crystals showed paramagnetic behavior with FIM long-range and AFM short-range interactions and a significant effect of orbital diamagnetism and Van Vleck’s paramagnetism. The electrical studies revealed a residual electrical conductivity without an intrinsic region and a change of sign of the Seebeck coefficient at ∼230 K, constant relative permittivity (∼8) and loss tangent (∼0.01) both up to 400 K and up to 1 MHz, as well as the Fermi energy (∼0.04 eV) and the Fermi temperature (∼500 K) determined from the diffusion component of the thermopower characteristic of the presence of shallow acceptor and donor levels. Compared to microcrystalline samples of the same chemical formula, a weakening of electron transport was observed in the studied single crystals due to the lack of thermal activation up to 400 K. The Czochralski method, however, had no significant effect on their magnetic and dielectric properties. The results presented in our paper make the CMNMWO single crystals attractive candidates for optical sensors as well as thermoelectric devices and lossless capacitors.

## Supporting Information Available

CIF files for CMNMWO single crystals: gron17_camnnd1_3.cif (CIF), gron17_camnnd2_1.cif (CIF)
Crystal data and structure refinement, bond lengths and angles, and anisotropic displacement parameters for both CMNMWO single crystals (PDF)

## Supplementary Material

ao3c05253_si_001.cif

ao3c05253_si_002.cif
