# Supplementary material for: Magnetic and Electrical Characteristics of Nd3+ and Mn2+-Co-doped Calcium Molybdato-Tungstates Single Crystals
Source: ACS Omega. 2023 Oct 2;8(41):38459–68. doi: 10.1021/acsomega.3c05253 (PMC10586187; doi:10.1021/acsomega.3c05253)
Supplement: Supplementary file 3 — ao3c05253_si_003.pdf [file ao3c05253_si_003.pdf]

Magnetic and Electrical Characteristics of Nd^{3+} and Mn^{2+}-co-doped calcium molybdato-tungstates single crystals

Bogdan Sawicki,[1] Elżbieta Tomaszewicz,*[2] Tadeusz Groń,[1] Monika Oboz,[1] Joachim Kusz[1] and Marek Berkowski[3]

[1] Institute of Physics, University of Silesia, 40-007 Katowice, Poland

[2] Faculty of Chemical Technology and Engineering, Department of Inorganic and Analytical Chemistry, West Pomeranian University of Technology, 71-065 Szczecin, Poland

[3] Institute of Physics, PAS, 02-668 Warszawa, Poland

* Corresponding author: tomela@zut.edu.pl

CIF files for CMNMWO single crystals have been deposited in the CCDC database with the following Nos. 2264487 and 2264488 as well as they have been attached as the following files: gron17_camnnd1_3.cif and gron17_camnnd2_1.cif.

Table S1. Crystal data and structure refinement for CMNMWO single crystal (*x* = 0.0050; *y* = 0.0050).

Empirical formula	$Ca_{0.98}Mn_{0.005}Nd_{0.01}(MoO_4)_{0.985}(WO_4)_{0.015}$	
Formula weight	202.26	
Temperature	293(1) K	
Wavelength	0.71073 Å	
Crystal system	Tetragonal	
Space group	I 4_1/a	
Unit cell dimensions	a = 5.22766(10) Å	α= 90°
	b = 5.22766(10) Å	β= 90°.
	c = 11.4330(3) Å	γ= 90°
Volume	312.447(14) $Å^3$	
Z	4	
Density (calculated)	4.300 Mg/m^3	
Absorption coefficient	6.299 mm^{-1}	
F(000)	379	
Crystal size	0.150 x 0.110 x 0.040 mm^3	
Theta range for data collection	4.287 to 40.140°	
Index ranges	-9<=h<=7, -9<=k<=8, -20<=l<=17	
Reflections collected	2720	
Independent reflections	481 [R(int) = 0.0175]	
Completeness to theta = 25.242°	100.0 %	
Absorption correction	Semi-empirical from equivalents	
Max. and min. transmission	1.00000 and 0.55492	
Refinement method	Full-matrix least-squares on F^2	
Data / restraints / parameters	481/0/15	
Goodness-of-fit on F^2	1.130	
Final R indices [I>2sigma(I)]	R1 = 0.0119, wR2 = 0.0315	
R indices (all data)	R1 = 0.0125, wR2 = 0.0320	
Extinction coefficient	0.115(3)	
Largest diff. peak and hole	0.399 and -0.915 (e $Å^{-3}$)	

Table S2. Atomic coordinates (×10^4) and equivalent isotropic displacement parameters ($Å^2$×10^3) for CMNMWO single crystal (*x* = 0.0050; *y* = 0.0050). U(eq) is defined as one third of the trace of the orthogonalized U^{ij} tensor.

Element	x	y	z	U(eq)
CaMn/Nd	0	2500	1250	9(1)
O	1483(1)	-66(1)	2906(1)	11(1)
Mo/W	0	2500	6250	7(1)

Table S3. Bond lengths [Å] and angles [°] for CMNMWO single crystal (*x* = 0.0050; *y* = 0.0050).

Interatomic distances (Å)					
Ca/Mn/Nd-O#1	2.4465(6)	Ca/Mn/Nd -O#5	2.4723(6)	Ca/Mn/Nd –Mo/W#10	3.69652(6)
Ca/Mn/Nd -O#2	2.4465(6)	Ca/Mn/Nd -O#6	2.4723(6)	Ca/Mn/Nd –Mo/W#11	3.69652(7)
Ca/Mn/Nd -O#3	2.4465(6)	Ca/Mn/Nd -O#7	2.4723(6)	O-Mo/W#12	1.7749(6)
Ca/Mn/Nd -O	2.4465(6)	Ca/Mn/Nd –Mo/W#8	3.69652(6)		
Ca/Mn/Nd -O#4	2.4723(6)	Ca/Mn/Nd –Mo/W#9	3.69652(6)		

Angles (°)					
O#1-Ca-O#2	126.792(18)	O#5-Ca-Mo#8	155.455(14)	Mo#12-O-Ca	132.49(3)
O#1-Ca-O#3	78.59(3)	O#6-Ca-Mo#8	98.175(14)	Mo#12-O-Ca#4	120.10(3)
O#2-Ca-O#3	126.792(18)	O#7-Ca-Mo#8	81.825(14)	Ca-O-Ca#4	103.89(2)
O#1-Ca-O	126.791(18)	O#1-Ca-Mo#9	52.281(15)	O#13-Mo-O#14	107.191(19)
O#2-Ca-O	78.59(3)	O#2-Ca-Mo#9	99.425(13)	O#13-Mo-O#12	114.13(4)
O#3-Ca-O	126.792(18)	O#3-Ca-Mo#9	127.719(15)	O#14-Mo-O#12	107.191(19)
O#1-Ca-O#4	151.87(2)	O-Ca-Mo#9	80.575(13)	O#13-Mo-O#15	107.191(19)
O#2-Ca-O#4	68.653(14)	O#4-Ca-Mo#9	155.455(14)	O#14-Mo-O#15	114.13(4)
O#3-Ca-O#4	73.876(12)	O#5-Ca-Mo#9	24.545(14)	O#12-Mo-O#15	107.191(19)
O-Ca-O#4	76.11(2)	O#6-Ca-Mo#9	81.825(14)	O#13-Mo-Ca#16	35.353(19)
O#1-Ca-O#5	73.876(12)	O#7-Ca-Mo#9	98.175(14)	O#14-Mo-Ca#16	101.42(2)
O#2-Ca-O#5	76.11(2)	Mo#8-Ca-Mo#9	180.0	O#12-Mo-Ca#16	144.647(19)
O#3-Ca-O#5	151.87(2)	O#1-Ca-Mo#10	80.576(13)	O#15-Mo-Ca#16	78.58(2)
O-Ca-O#5	68.652(14)	O#2-Ca-Mo#10	52.281(15)	O#13-Mo-Ca#17	144.647(19)
O#4-Ca-O#5	134.05(3)	O#3-Ca-Mo#10	99.424(13)	O#14-Mo-Ca#17	78.58(2)
O#1-Ca-O#6	76.11(2)	O-Ca-Mo#10	127.718(15)	O#12-Mo-Ca#17	35.353(19)
O#2-Ca-O#6	151.87(2)	O#4-Ca-Mo#10	98.175(14)	O#15-Mo-Ca#17	101.42(2)
O#3-Ca-O#6	68.653(14)	O#5-Ca-Mo#10	81.825(14)	Ca#16-Mo-Ca#17	180.0
O-Ca-O#6	73.876(12)	O#6-Ca-Mo#10	155.455(14)	O#13-Mo-Ca#18	78.58(2)
O#4-Ca-O#6	98.763(10)	O#7-Ca-Mo#10	24.545(14)	O#14-Mo-Ca#18	35.353(19)
O#5-Ca-O#6	98.763(10)	Mo#8-Ca-Mo#10	90.0	O#12-Mo-Ca#18	101.42(2)
O#1-Ca-O#7	68.653(14)	Mo#9-Ca-Mo#10	90.0	O#15-Mo-Ca#18	144.647(19)
O#2-Ca-O#7	73.876(12)	O#1-Ca-Mo#11	99.424(13)	Ca#16-Mo-Ca#18	90.0
O#3-Ca-O#7	76.11(2)	O#2-Ca-Mo#11	127.719(15)	Ca#17-Mo-Ca#18	90.0
O-Ca-O#7	151.87(2)	O#3-Ca-Mo#11	80.576(13)	O#13-Mo-Ca#19	101.42(2)
O#4-Ca-O#7	98.763(10)	O-Ca-Mo#11	52.282(15)	O#14-Mo-Ca#19	144.647(19)
O#5-Ca-O#7	98.763(10)	O#4-Ca-Mo#11	81.825(14)	O#12-Mo-Ca#19	78.58(2)
O#6-Ca-O#7	134.05(3)	O#5-Ca-Mo#11	98.175(14)	O#15-Mo-Ca#19	35.353(19)
O#1-Ca-Mo#8	127.719(15)	O#6-Ca-Mo#11	24.545(14)	Ca#16-Mo-Ca#19	90.0
O#2-Ca-Mo#8	80.575(13)	O#7-Ca-Mo#11	155.455(14)	Ca#17-Mo-Ca#19	90.0
O#3-Ca-Mo#8	52.281(15)	Mo#8-Ca-Mo#11	90.0	Ca#18-Mo-Ca#19	180.0
O-Ca-Mo#8	99.425(13)	Mo#9-Ca-Mo#11	90.0		
O#4-Ca-Mo#8	24.545(14)	Mo#10-Ca-Mo#11	180.0		

Symmetry transformations used to generate equivalent atoms:
#1 y-1/4,-x+1/4,-z+1/4 #2 -x+0,-y+1/2,z+0 #3 -y+1/4,x+1/4,-z+1/4
#4 -x+1/2,-y+1/2,-z+1/2 #5 x-1/2,y,-z+1/2 #6 -y+1/4,x-1/4,z-1/4
#7 y-1/4,-x+3/4,z-1/4 #8 x+1/2,y+1/2,z-1/2 #9 x-1/2,y-1/2,z-1/2
#10 x-1/2,y+1/2,z-1/2 #11 x+1/2,y-1/2,z-1/2
#12 -x,-y,-z+1 #13 x,y+1/2,-z+1 #14 -y-1/4,x+1/4,z+1/4
#15 y+1/4,-x+1/4,z+1/4 #16 x+1/2,y+1/2,z+1/2
#17 x-1/2,y-1/2,z+1/2 #18 x-1/2,y+1/2,z+1/2
#19 x+1/2,y-1/2,z+1/2

Table S4. Anisotropic displacement parameters (Å^2×10^3) for CMNMWO single crystal (*x* = 0.0050; *y* = 0.0050). The anisotropic displacement factor exponent takes the form: $-2\pi^2[h^2a^{*2}U^{11} + ... + 2\ h\ k\ a^*\ b^*\ U^{12}]$.

Element	U^{11}	U^{22}	U^{33}	U^{23}	U^{13}	U^{12}
CaMn/Nd	9(1)	9(1)	8(1)	0	0	0
O	11(1)	11(1)	11(1)	2(1)	-1(1)	-2(1)
Mo/W	6(1)	6(1)	8(1)	0	0	0

Table S5. Crystal data and structure refinement for CMNMWO single crystal (x = 0.0098; y = 0.0050).

Empirical formula	$Ca_{0.9653}Mn_{0.0050}Nd_{0.0198}(MoO_4)_{0.9703}(WO_4)_{0.0297}$	
Formula weight	204.42	
Temperature	293(1) K	
Wavelength	0.71073 Å	
Crystal system	Tetragonal	
Space group	I 4_1/a	
Unit cell dimensions	a = 5.23207(12) Å	α= 90°
	b = 5.23207(12) Å	β= 90°.
	c = 11.4424(5) Å	γ= 90°
Volume	313.23(2) Å^3	
Z	4	
Density (calculated)	4.335 Mg/m^3	
Absorption coefficient	6.911 mm^{-1}	
F(000)	382	
Crystal size	0.070 x 0.060 x 0.040 mm^3	
Theta range for data collection	4.283 to 40.664°.	
Index ranges	-9<=h<=8, -8<=k<=9, -20<=l<=17	
Reflections collected	2928	
Independent reflections	505 [R(int) = 0.0251]	
Completeness to theta = 25.242°	100.0 %	
Absorption correction	Semi-empirical from equivalents	
Max. and min. transmission	1.00000 and 0.67475	
Refinement method	Full-matrix least-squares on F^2	
Data / restraints / parameters	505/0/15	
Goodness-of-fit on F^2	1.100	
Final R indices [I>2sigma(I)]	R1 = 0.0151, wR2 = 0.0370	
R indices (all data)	R1 = 0.0190, wR2 = 0.0386	
Extinction coefficient	0.0249(11)	
Largest diff. peak and hole	0.667 and -0.691 (e Å^{-3})	

Table S6. Atomic coordinates (×10^4) and equivalent isotropic displacement parameters (Å^2×10^3) for CMNMWO single crystal (x = 0.0098; y = 0.0050). U(eq) is defined as one third of the trace of the orthogonalized U^{ij} tensor.

Element	x	y	z	U(eq)
Ca/Mn/Nd	0	2500	1250	9(1)
O	1482(2)	-68(2)	2904(1)	12(1)
Mo/W	0	2500	6250	7(1)

Table S7. Bond lengths [Å] and angles [°] for CMNMWO single crystal (*x* = 0.0098; *y* = 0.0050).

Interatomic distances (Å)					
Ca/Mn/Nd-O#1	2.4473(9)	Ca/Mn/Nd -O#5	2.4757(8)	Ca/Mn/Nd –Mo/W#10	3.69963(8)
Ca/Mn/Nd -O#2	2.4473(9)	Ca/Mn/Nd -O#6	2.4757(8)	Ca/Mn/Nd –Mo/W#11	3.69963(8)
Ca/Mn/Nd -O#3	2.4473(9)	Ca/Mn/Nd -O#7	2.4757(8)	O-Mo/W#12	1.7768(8)
Ca/Mn/Nd -O	2.4473(9)	Ca/Mn/Nd –Mo/W#8	3.69963(8)		
Ca/Mn/Nd -O#4	2.4757(8)	Ca/Mn/Nd –Mo/W#9	3.69963(8)		

Angles (°)					
O#1-Ca-O#2	126.75(3)	O#5-Ca-Mo#8	155.43(2)	Mo#12-O-Ca	132.54(4)
O#1-Ca-O#3	78.66(4)	O#6-Ca-Mo#8	98.17(2)	Mo#12-O-Ca#4	120.02(4)
O#2-Ca-O#3	126.75(3)	O#7-Ca-Mo#8	81.83(2)	Ca-O-Ca#4	103.89(3)
O#1-Ca-O	126.75(3)	O#1-Ca-Mo#9	52.25(2)	O#13-Mo-O#14	107.25(3)
O#2-Ca-O	78.66(4)	O#2-Ca-Mo#9	99.446(19)	O#13-Mo-O#12	114.01(6)
O#3-Ca-O	126.75(3)	O#3-Ca-Mo#9	127.75(2)	O#14-Mo-O#12	107.25(3)
O#1-Ca-O#4	151.93(4)	O-Ca-Mo#9	80.554(19)	O#13-Mo-O#15	107.25(3)
O#2-Ca-O#4	68.62(2)	O#4-Ca-Mo#9	155.43(2)	O#14-Mo-O#15	114.01(6)
O#3-Ca-O#4	73.876(17)	O#5-Ca-Mo#9	24.57(2)	O#12-Mo-O#15	107.25(3)
O-Ca-O#4	76.11(3)	O#6-Ca-Mo#9	81.83(2)	O#13-Mo-Ca#16	35.41(3)
O#1-Ca-O#5	73.876(17)	O#7-Ca-Mo#9	98.17(2)	O#14-Mo-Ca#16	101.42(3)
O#2-Ca-O#5	76.11(3)	Mo#8-Ca-Mo#9	180.0	O#12-Mo-Ca#16	144.59(3)
O#3-Ca-O#5	151.93(4)	O#1-Ca-Mo#10	80.554(19)	O#15-Mo-Ca#16	78.58(3)
O-Ca-O#5	68.62(2)	O#2-Ca-Mo#10	52.25(2)	O#13-Mo-Ca#17	144.59(3)
O#4-Ca-O#5	133.98(4)	O#3-Ca-Mo#10	99.446(19)	O#14-Mo-Ca#17	78.58(3)
O#1-Ca-O#6	76.11(3)	O-Ca-Mo#10	127.75(2)	O#12-Mo-Ca#17	35.41(3)
O#2-Ca-O#6	151.93(4)	O#4-Ca-Mo#10	98.17(2)	O#15-Mo-Ca#17	101.42(3)
O#3-Ca-O#6	68.62(2)	O#5-Ca-Mo#10	81.83(2)	Ca#16-Mo-Ca#17	180.0
O-Ca-O#6	73.877(17)	O#6-Ca-Mo#10	155.43(2)	O#13-Mo-Ca#18	78.58(3)
O#4-Ca-O#6	98.787(15)	O#7-Ca-Mo#10	24.57(2)	O#14-Mo-Ca#18	35.41(3)
O#5-Ca-O#6	98.787(15)	Mo#8-Ca-Mo#10	90.0	O#12-Mo-Ca#18	101.42(3)
O#1-Ca-O#7	68.62(2)	Mo#9-Ca-Mo#10	90.0	O#15-Mo-Ca#18	144.59(3)
O#2-Ca-O#7	73.876(17)	O#1-Ca-Mo#11	99.446(19)	Ca#16-Mo-Ca#18	90.0
O#3-Ca-O#7	76.11(3)	O#2-Ca-Mo#11	127.75(2)	Ca#17-Mo-Ca#18	90.0
O-Ca-O#7	151.93(4)	O#3-Ca-Mo#11	80.554(19)	O#13-Mo-Ca#19	101.42(3)
O#4-Ca-O#7	98.787(15)	O-Ca-Mo#11	52.25(2)	O#14-Mo-Ca#19	144.59(3)
O#5-Ca-O#7	98.787(15)	O#4-Ca-Mo#11	81.83(2)	O#12-Mo-Ca#19	78.58(3)
O#6-Ca-O#7	133.98(4)	O#5-Ca-Mo#11	98.17(2)	O#15-Mo-Ca#19	35.41(3)
O#1-Ca-Mo#8	127.75(2)	O#6-Ca-Mo#11	24.57(2)	Ca#16-Mo-Ca#19	90.0
O#2-Ca-Mo#8	80.554(19)	O#7-Ca-Mo#11	155.43(2)	Ca#17-Mo-Ca#19	90.0
O#3-Ca-Mo#8	52.25(2)	Mo#8-Ca-Mo#11	90.0	Ca#18-Mo-Ca#19	180.0
O-Ca-Mo#8	99.446(19)	Mo#9-Ca-Mo#11	90.0		
O#4-Ca-Mo#8	24.57(2)	Mo#10-Ca-Mo#11	180.0		

Symmetry transformations used to generate equivalent atoms:
#1 y-1/4,-x+1/4,-z+1/4 #2 -x+0,-y+1/2,z+0 #3 -y+1/4,x+1/4,-z+1/4
#4 -x+1/2,-y+1/2,-z+1/2 #5 x-1/2,y,-z+1/2 #6 -y+1/4,x-1/4,z-1/4
#7 y-1/4,-x+3/4,z-1/4 #8 x+1/2,y+1/2,z-1/2 #9 x-1/2,y-1/2,z-1/2
#10 x-1/2,y+1/2,z-1/2 #11 x+1/2,y-1/2,z-1/2
#12 -x,-y,-z+1 #13 x,y+1/2,-z+1 #14 -y-1/4,x+1/4,z+1/4
#15 y+1/4,-x+1/4,z+1/4 #16 x+1/2,y+1/2,z+1/2
#17 x-1/2,y-1/2,z+1/2 #18 x-1/2,y+1/2,z+1/2

#19 x+1/2,y-1/2,z+1/2

Table S8. Anisotropic displacement parameters (Å^2×10^3) for CMNMWO single crystal (*x* = 0.0098; *y* = 0.0050). The anisotropic displacement factor exponent takes the form: $-2\pi^2[h^2a^{*2}U^{11} + ... + 2 h k a^* b^* \cdot U^{12}]$.

Element	U^{11}	U^{22}	U^{33}	U^{23}	U^{13}	U^{12}
Ca/Mn/Nd	10(1)	10(1)	9(1)	0	0	0
O	11(1)	12(1)	12(1)	2(1)	-1(1)	-2(1)
Mo/W	7(1)	7(1)	9(1)	0	0	0
